# Prediction of vascular abnormalities on CT angiography in patients with acute headache

**DOI:** 10.1002/brb3.997

**Published:** 2018-05-09

**Authors:** Imanda M. E. Alons, Ben F. J. Goudsmit, Korné Jellema, Marianne A. A. van Walderveen, Marieke J. H. Wermer, Ale Algra

**Affiliations:** ^1^ Department of Neurology MCH Westeinde The Hague The Netherlands; ^2^ Department of Neurology LUMC Leiden The Netherlands; ^3^ Department of Radiology LUMC Leiden The Netherlands; ^4^ Department of Clinical Epidemiology LUMC Leiden The Netherlands; ^5^ Department of Neurology and Neurosurgery Brain Center Rudolph Magnus UMC Utrecht Utrecht The Netherlands; ^6^ Julius Center for Health Sciences and Patient Care UMC Utrecht Utrecht The Netherlands

**Keywords:** acute headache, CT angiography, neuro‐imaging, prediction model, subarachnoid hemorrhage, thunderclap headache, vascular abnormality

## Abstract

**Objectives:**

Patients with acute headache increasingly undergo CT‐angiography (CTA) to evaluate underlying vascular causes. The aim of this study is to determine clinical and non‐contrast CT (NCCT) criteria to select patients who might benefit from CTA.

**Methods:**

We retrospectively included patients with acute headache who presented to the emergency department of an academic medical center and large regional teaching hospital and underwent NCCT and CTA. We identified factors that increased the probability of finding a vascular abnormality on CTA, performed multivariable regression analyses and determined discrimination with the c‐statistic.

**Results:**

A total of 384 patients underwent NCCT and CTA due to acute headache. NCCT was abnormal in 194 patients. Among these, we found abnormalities in 116 cases of which 99 aneurysms. In the remaining 190 with normal NCCT we found abnormalities in 12 cases; four unruptured aneurysms, three cerebral venous thrombosis’, two reversible cerebral vasoconstriction syndromes, two cervical arterial dissections and one cerebellar infarction. In multivariable analysis abnormal NCCT, lowered consciousness and presentation within 6 hr of headache onset were independently associated with abnormal CTA. The c‐statistic of abnormal NCCT alone was 0.80 (95% CI: 0.75–0.80), that also including the other two variables was 0.84 (95% CI: 0.80–0.88). If NCCT was normal no other factors could help identify patients at risk for abnormalities.

**Conclusions:**

In patients with acute headache abnormal NCCT is the strongest predictor of a vascular abnormality on CTA. If NCCT is normal no other predictors increase the probability of finding an abnormality on CTA and diagnostic yield is low.

## 1 INTRODUCTION

Acute headache may have several vascular causes including subarachnoid hemorrhage (SAH) from a ruptured aneurysm, cerebral venous thrombosis (CVT), reversible cerebral vasoconstriction syndrome (RCVS) or arterial dissection (Cumurciuc, Crassard, Sarov, Valade, & Bousser, 2005; Day & Raskin, 1986; Ducros, 2012; Maruyama et al., 2012; McCarron & Choudhari, 2005; Mitsias & Ramadan, 1992; Raps et al., 1993; Witham & Kaufmann, 2000). These vascular abnormalities can be diagnosed in the emergency setting with CT angiography (CTA).

An additional CTA causes excess radiation exposure of 2.5 mSv after normal NCCT (which also causes 2.5 mSv; Cohnen et al., 2006). The iodine contrast which is given intravenously may cause allergic reactions and contrast nephropathy in a small number of cases (Cohnen et al., 2006; Dittrich et al., 2007; Luitse et al., 2015). Therefore, discriminating clinical characteristics such as abnormalities on neurological examination to more selectively apply CTA would be valuable for physicians in reducing unnecessary diagnostic tests.

Previous research focused on the value of CTA of the brain and cervical arteries in all emergency department patients (Jamshidi et al., 2011) or on development of a clinical decision rule for diagnosing SAH, regardless of CTA results, in patients with acute headache (Perry et al., 2013). These studies found that several characteristics such as signs of hemorrhage on non‐contrast head CT (NCCT), deficit on neurological examination, acute onset of headache (Jamshidi et al., 2011), age over 40 years, nuchal rigidity or onset during exertion (Perry et al., 2013) are associated with a higher risk of abnormalities on CTA or the chance of SAH. A clinical prediction rule for vascular abnormalities on CTA for patients in the emergency department with acute headache and a normal NCCT is currently not available.

In this study, we evaluated patients presenting with acute headache in whom CTA was performed to exclude secondary forms of headache. We aimed to develop a diagnostic prediction model with headache, patient, and NCCT characteristics to identify patients with the highest probability of an abnormality on CTA.

## 2 MATERIALS AND METHODS

We retrospectively evaluated all patients who underwent a cerebral and cervical CTA between January 2011 and December 2014 in the emergency department of the Leiden University Hospital (LUMC), a tertiary vascular neurology referral center and university teaching hospital, and the Haaglanden Medical Centre in The Hague, a tertiary vascular referral center and a secondary teaching hospital. We included all patients who presented with acute headache. We defined acute headache as headache peaking within 5 min and lasting more than 1 hr. We excluded patients who were comatose (EMV < 9), who had an ongoing seizure or were otherwise unable to express whether they had headache. Patients were also excluded when their headache developed after a trauma or after start of focal neurological deficits.

The patients were identified in the hospital digital medical chart system by evaluating all patients who received a cerebral and cervical CTA and were admitted to the emergency department. All medical charts were reviewed for the in‐ and exclusion criteria. Chart review was done by IMA and BFJG with a standardized data abstraction form. We charted patient characteristics (age, sex, patient history and medication, focal neurological deficits, seizures, loss of consciousness ongoing or of short duration not deemed a seizure, nausea, vomiting, nuchal rigidity, papillary edema) and headache characteristics (location, duration, presence of aura, autonomous symptoms).

In the Leiden University Hospital patients were scanned from the aortic arch up to the vertex with an Aquilion One (Aquilion ONE; Toshiba Medical Systems, Tokyo, Japan) or Aquilion 64 CT scanner (Aquilion 64; Toshiba Medical Systems, Otawara, Japan). In the MC Haaglanden patients were scanned from the aortic arch to the vertex with a General Electrics Lightspeed 64‐slice scanner (General Electric, Chicago IL, USA).

We recorded adverse events of the CTA including allergic reactions, kidney failure or infections after IV catheter use. The adverse events were deducted from the authorized hospital form used to chart any clinical adverse events during admission.

The study was evaluated and approved by the local ethics committee, which waived the need for patient consent as all data were recorded anonymously.

### 2.1 Statistical analysis

We calculated prevalence ratios with corresponding 95% confidence intervals for patient characteristics in relation to presence of abnormalities at CTA. Multivariable logistic regression analysis was performed with a vascular abnormality at CTA as the outcome variable. Candidate predictors were considered for entrance into multivariable regression models irrespective of their univariable association with a vascular abnormality at CTA (Royston, Moons, Altman, & Vergouwe, 2009). We included all candidate predictors in the multivariable logistic regression model and excluded them stepwise when the likelihood ratio test had *p* > .15. The discriminative performance, to which the prognostic model enables discrimination between patients with and without vascular abnormalities at CTA, was described by the c‐statistic. The c‐statistic varies between .5 (a non‐informative model) and 1.0 (a perfect model; Greving et al., 2012). Our report on prediction adheres to the TRIPOD (Transparent reporting of a multivariable prediction model for individual prognosis or diagnosis) guidelines (Collins, Reitsma, Altman, & Moons, 2015). This guideline consists of a 22 item checklist detailing the essential information that should be included in a report of a prediction modeling study.

Some variables, such as papillary edema or nuchal rigidity, were not recorded and thus missing in some cases. The absence of these clinical signs in the examination was also evaluated to ascertain whether this was a predictive factor.

## 3 RESULTS

We included 384 patients (flowchart 1). The average age of the participants was 51 years and 150 (39.1%) were men. In the entire group of patients 129 presented with a normal neurological examination (33.6%), 190 had a normal NCCT (49.4%), and 128 had an abnormality on CTA (33.3%). Other characteristics can be found in Table 1.

**Table 1:** Characteristics of the 384 included patients

Characteristic	No. (%)
Age (years)	51; SD 15
Male sex	150 (39.1%)
Duration to presentation (days ave)	2.4; SD 4.9
Headache location	
Halfsided	30 (7.8%)
Whole head	161 (41.6%)
Localized	193 (50.3%)
Nausea	97 (25.1%)
Vomiting	186 (48.4%)
Neurological examination	
Normal	129 (33.6%)
Abnormal	255 (66.4%)
Subjective deficit	97 (25.3%)
Motor lateralization	42 (10.9%)
Sensory lateralization	29 (7.5%)
Papillary edema	6 (0.1%)
Abnormal pupils	23 (5.9%)
Nuchal rigidity	44 (11.5%)
NCCT	
Normal	190 (49.4%)
SAH	128 (33.1%)
Intraparenchymal hemorrhage	32 (8.3%)
Ischemia	11 (2.8%)
Space occupying lesion	10 (2.6%)
Sinusitis	7 (1.8%)
SDH	2 (0.5%)
Other	4 (1%)
CTA	
Normal	256 (66.7%)
Aneurysm	103 (26.8%)
CVT	6 (1.6%)
AVM	7 (1.8%)
Dissection	5 (1.3%)
Ischemia	3 (0.7%)
RCVS	2 (0.5%)
AV fistula	2 (0.5%)
LP	
Not performed	287 (74.7%)
Normal	69 (18%)
Hemorrhage	14 (3.6%)
Meningitis	10 (2.6%)
Raised pressure	4 (1%)

AVM, arterio‐venous malformation; CTA, CT angiography; CVT, cerebral venous thrombosis; LP, lumbar puncture; NCCT, non contrast CT; RCVS, reversible cerebral vasoconstriction syndrome; SAH, subarachnoid hemorrhage; SDH, subdural hematoma.

### 3.1 Patients with abnormal NCCT

**Figure 1:**
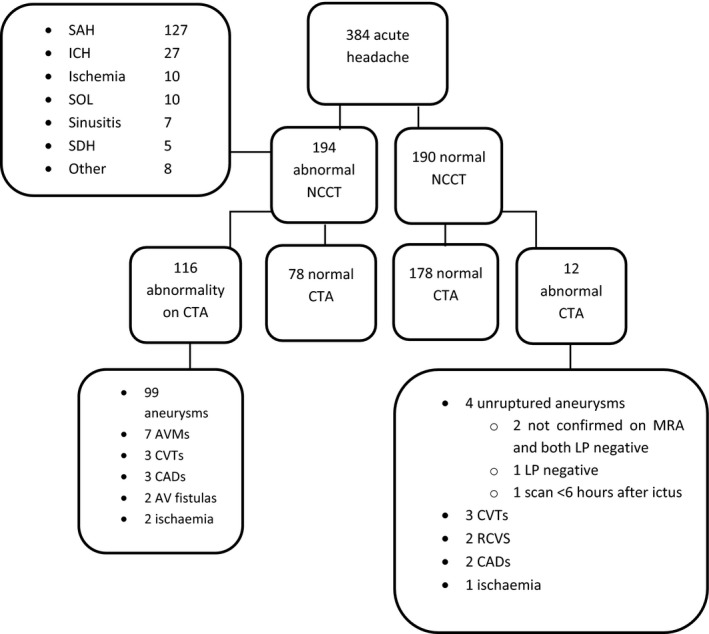
Patient inclusion flowchart. ED, emergency department; NCCT, non‐contrast CT; CTA, CT angiography; CVT, cerebral venous thrombosis; AVM, arteriovenous malformation; LP, Lumbar puncture; RCVS, reversible cerebral vasoconstriction syndrome; CAD, cervical arterial dissection; IH, intraparenchymal hemorrhage; SOL space occupying lesion

Of the 194 patients with an abnormal NCCT there were 116 with an abnormality on CTA (59.8%; Figure 1). All these abnormalities were symptomatic and were treated accordingly. About 128 (74.1%) patients had SAH on NCCT and in 96 (95.1%) of these patients an aneurysm was found. In two patients the CTA showed no abnormalities but an aneurysm was detected after MRI and digital subtraction angiography. Three patients with an intraparenchymal hemorrhage also had a causative aneurysm on CTA. The number needed to scan to find a clinically relevant vascular abnormality on CTA in this group was less than two patients (1.7).

### 3.2 Patients with normal NCCT

In 190 patients (49.5%) the NCCT was normal. Twelve (6.3%) of these patients had a vascular abnormality on CTA and in seven (3.7%) of these patients the abnormality was thought to be the cause of the headache. These seven abnormalities were also of clinical significance because they warranted a change of medication or more intensified follow up. We could not define whether the found aneurysms were relevant because we did not have sufficient data to retrieve if they had ruptured or not. Unruptured aneurysms are not a likely cause of headache and may be an incidental finding. Also data on the necessity of treatment according to our guidelines were not completely available. Three patients had CVT, one of which had been diagnosed earlier and was unchanged; we did not count this as a relevant finding. Two patients were diagnosed with RCVS, two with a cervical arterial dissection and one with a cerebellar infarction. Thus, the number needed to scan to find a clinically relevant abnormality was 27.

In addition to the clinically relevant findings, unruptured aneurysms were found in four patients; in two patients the aneurysms could not be confirmed on MRI, one patient with an MRI confirmed aneurysm had a normal LP and the fourth patient did not receive an LP but presented within 6 hr on the ED, with normal NCCT ruling out SAH.

### 3.3 Multivariable analyses

In the univariable analyses of all 384 patients there were 14 variables associated with either CTA abnormalities or normal CTA. Age, presentation within 6 hr of headache, vomiting, collapse, impaired consciousness, nuchal rigidity, subjective neurological deficit, abnormal pupillary responses, motor deficit were associated with abnormal CTA. Normal NCCT, unilateral headache, normal neurological examination, normal Glasgow Coma Score and a history of previous headaches were associated with normal CTA (Table 2).

**Table 2:** Abnormality at CTA in relation to patient characteristics in 384 included patients

Patient characteristics	% abnormal CTA		
	Baseline characteristic present, *n*/*N*(%)	Baseline characteristic absent, *n*/*N*(%)	Prevalence ratio^b^ (95% CI)
Age 45–65 years	71/183 (38.8%)	21/119 (17.6%)^a^	2.20 (1.43–3.38)
Age ≥65 years	36/82 (43.9%)	21/119 (17.6%)^a^	2.49 (1.57–3.94)
Female sex	79/234 (33.8%)	49/150 (32.7%)	1.03 (0.77–1.38)
Presentation <6 hr	83/179 (46.4%)	44/204 (21.6%)	2.15 (1.58–2.92)
Unilateral headache	3/30 (10.0%)	125/354 (35.3%)	0.28 (0.10–0.84)
Aura	3/21 (14.3%)	84/296 (28.4%)	0.50 (0.17–1.46)
No Aura recorded	41/67 (61.2%)	87/317 (27.4%)	2.23 (1.72–2.90)
Vomiting	38/186 (20.4%)	76/172 (44.2%)	0.46 (0.33–0.64)
Seizure	3/8 (37.5%)	125/376 (33.2%)	1.13 (0.46–2.79)
Collapse	12/23 (52.2%)	116/361 (32.1%)	1.62 (1.07–2.47)
Altered mental state	4/7 (57.1%)	124/377 (32.9%)	1.74 (0.90–3.35)
Impaired consciousness	24/33 (72.7%)	104/351 (29.6%)	2.45 (1.89–3.20)
Normal glascow coma scale	82/309 (26.5%)	46/75 (61.3%)	0.43 (0.33–0.56)
Nuchal rigidity	25/44 (56.8%)	73/291 (25.1%)	2.26 (1.64–3.14)
Subjective neurological deficit	39/97 (40.2%)	84/282 (29.8%)	1.35 (1.00–1.83)
Pupillary responses abnormal	14/24 (58.3%)	109/353 (30.9%)	1.89 (1.30–2.74)
Motor deficit	19/42 (45.2%)	105/338 (31.1%)	1.46 (1.01–2.11)
Sensory deficit	10/29 (34.5%)	113/350 (32.3%)	1.07 (0.63–1.80)
Neurological examination normal	22/129 (17.1%)	106/255 (41.6%)	0.41 (0.27–0.62)
Non contrast CT normal	12/190 (6.3%)	116/194 (59.8%)	0.11 (0.06–0.18)
Lumbar puncture normal	6/69 (8.7%)	6/29 (20.7%)	0.42 (0.15–1.20)
Lumbar puncture hemorrhage	4/14 (28.6%)	8/84 (9.5%)	3.00 (1.04–8.65)
Lumbar puncture infectious	1/10 (10%)	11/88 (12.5%)	0.80 (0.12–5.67)
Lumbar puncture raised pressure	1/4 (25%)	11/94 (11.7%)	2.14 (0.36–12.74)
Neurological history	17/46 (37.0%)	106/333 (31.8%)	1.16 (0.77–1.75)
Headache history	19/82 (23.2%)	106/299 (35.5%)	0.65 (0.43–1.00)

a Age <45 years was taken as a reference.

b Prevalence ratio is the proportion of patients with a vascular abnormality with the given baseline characteristic over the proportion in the group without that characteristic. If the ratio is <1 there are less vascular abnormalities in the group with the baseline characteristic, if the ratio is >1 there are more vascular abnormalities in that group.

In the multivariate analyses three factors were significantly associated with an abnormal CTA: presentation within 6 hr of headache, abnormal NCCT and ongoing impaired consciousness at time of the NCCT (Table 3). The c‐statistic of this model was .84 (95% CI: 0.80–0.88). A model based on clinical characteristics only contained seven variables (presentation <6 hr, half sided headache, aura, vomiting, nuchal rigidity, pupil abnormalities, and lumbar puncture performed) and had a c‐statistic of .80 (95% CI 0.76–0.85). A model based on NCCT alone had a c statistic of .80 (0.75–0.85). The difference between the c‐statistics was not statistically significant.

**Table 3:** Multivariable model on basis of clinical characteristics and NCCT

	Odds ratio	95% CI
Presentation <6 hr	1.82	1.06–3.12
Normal consciousness	0.46	0.24–0.86
Normal NCCT	0.06	0.03–0.11
c statistic	0.84	0.80–0.88

We attempted to assess which factors would be related to an abnormal CTA in the 190 patients with a normal NCCT. However, because there were only 12 patients with a CTA abnormality in this group we could not identify these factors as overall yield was low.

## 4 DISCUSSION

We found that the yield of CTA in patients with acute headache presenting at the emergency department and an abnormal NCCT is high. An abnormal NCCT was the strongest predictor for finding an abnormality on CTA. When combined with the two other clinical factors that contributed independently to finding an abnormal CTA, an impaired lowered consciousness and presentation within 6 hr of headache, discrimination was not better than when NCCT alone was used. In patients with normal NCCT the diagnostic yield was low and in only seven out of 190 patients a clinical relevant abnormality was found.

To the best of our knowledge, this is the first study focusing on a prediction model for finding an abnormality on CTA in acute headache focusing on a broader diagnostic range than just SAH. We included patients from an academic medical center and a large teaching hospital to improve the generalizability of our results.

Our study has several limitations. First, there is a likely selection bias in the performed CTAs limiting the number of CTAs in patients with normal NCCT or normal neurological examination. Due to the retrospective design of our study we do not know why the treating physician chose to perform these CTAs and more importantly, we cannot determine how many patients did not receive a CTA at all. Second, in some subgroups, such as patients with a normal NCCT, the number of abnormalities was small. Due to the limited number of abnormalities there was insufficient power to determine clinically relevant factors. Alternatively, one could reason that abnormalities in this group are so few that CTA scanning in these patients is not cost‐effective. Finally, we had to rely on retrospective chart review and some data, for instance on the presence of nuchal rigidity or evaluation of papillary edema, was missing in a large part of patients, because the performance of such neurological tests may very much depend on the severity of the clinical presentation.

Patients with acute headache and an abnormal NCCT should receive CTA. The yield of CTA in patients with a normal NCCT was low and the number needed to scan to find a clinically relevant abnormality was 27. We feel this may still warrant CTA in this group, particularly if high diagnostic sensitivity is strived for. Clinically relevant diseases such as RVCS or CVT may be missed without additional imaging of the vessels. A prospective study aimed at identifying criteria for selecting patients with a normal NCCT for CTA is therefore needed.

## CONFLICT OF INTEREST

On behalf of all authors, the corresponding author declares no conflicts of interest.
